# Taxonomical Evaluation of Plant Chloroplastic Markers by Bayesian Classifier

**DOI:** 10.3389/fpls.2021.782663

**Published:** 2022-02-03

**Authors:** Luisa Matiz-Ceron, Alejandro Reyes, Juan Anzola

**Affiliations:** ^1^Research Group in Computational Biology and Microbial Ecology, Department of Biological Sciences, Universidad de los Andes, Bogotá, Colombia; ^2^Max Planck Tandem Group in Computational Biology, Universidad de los Andes, Bogotá, Colombia; ^3^Department of Engineering and Natural Sciences, Universidad Central, Bogotá, Colombia

**Keywords:** Naïve Bayesian classifier, metabarcoding, *matK*, *trnL*, taxonomic classification, chloroplast

## Abstract

DNA barcodes are standardized sequences that range between 400 and 800 bp, vary at different taxonomic levels, and make it possible to assign sequences to species that have been previously taxonomically characterized. Several DNA barcodes have been postulated for plants, nonetheless, their classification potential has not been evaluated for metabarcoding, and as a result, it would appear as none of them excels above the others in this area. One tool that has been widely used and served as a baseline when evaluating new approaches is Naïve Bayesian Classifiers (NBC). The present study aims at evaluating the classification power of several plant chloroplast genetic markers that have been proposed as barcodes (*trnL*, *rpoB*, *rbcL*, *matK*, *psbA-trnH*, and *psbK*) using an NBC. We performed the classification at different taxonomic levels, and identified problematic genera when resolution was desired. We propose *matK* and *trnL* as potential candidate markers with resolution up to genus level. Some problematic genera within certain families could lead to the misclassification no matter which marker is used (i.e., *Aegilops*, *Gueldenstaedtia*, *Helianthus*, *Oryza*, *Shorea*, *Thysananthus*, and *Triticum*). Finally, we suggest recommendations for the taxonomic identification of plants in samples with potential mixtures.

## Introduction

In recent years, DNA barcoding has been proposed as a method to survey biodiversity in the field (Hebert et al., 2003; Gross, 2012). DNA barcodes were proposed originally for animal classification (Hebert et al., 2003), but later, they were proposed for plants as well (Kress and Erickson, 2007). DNA barcoding represents an efficient tool for the identification of cryptic or invasive species (Lopez-Vaamonde et al., 2021), conservation, and community ecology (Hollingsworth et al., 2011; Yessoufou et al., 2013; Bezeng et al., 2017). This tool is based on the conserved DNA biomarkers with more interspecific than intraspecific variability creating a barcoding gap (Čandek and Kuntner, 2015), which allows the possibility to identify an organism at different taxonomic levels. An appropriate marker must have the following characteristics: (a) a significant genetic diversity, based on the desired resolution of the barcode, with conserved flanking sites to enable primer design; (b) An appropriate size for DNA extraction and amplification protocols; and (c) to be as generalist as possible, it should be present in all the targeted taxons (Kress et al., 2005; CBOL Plant Working Group, 2009). In recent years, advances in sequencing technologies have opened the possibility of ecological surveys based on sequencing data. Examples of these are the *16S rRNA* gene in prokaryotes, the *Internal Transcribed Spacer* (*ITS*) region in fungi and the *Cytochrome Oxidase I* (*COI*) in animals. Despite these advances, there are several taxonomic groups for which no ideal marker has been found for classification purposes or metabarcoding analysis.

Given the low mutation rate of mitochondrial DNA, mitochondrial cytochrome oxidase I (*COI*) cannot be used in plants (CBOL Plant Working Group, 2009; Li et al., 2021). In consequence, extensive search within nuclear and chloroplast genomes have been performed to identify suitable regions for barcode design. Three main regions have been proposed, the *ribulose-1*,*5-bisphosphate carboxylase/oxygenase large subunit or RuBisCO large subunit* (*rbcL*), *maturase K* (*matK*), and *Internal Transcribed Spacer 2* (*ITS2*) (Cowan et al., 2006; CBOL Plant Working Group, 2009; China Plant BOL Group, 2011). However, none of them have the precision that *COI* displays for animals (Pang et al., 2012), they do not have sufficient resolution for groups, such as lichens, bryophytes, or ferns (Kress and Erickson, 2007), and are ineffective for samples with degraded or fragmented DNA (Mallott et al., 2018). In consequence, other regions, such as *Transfer RNA T—L spacer* (*trnL*), *photosystem II protein D1—Transfer RNA H* (*psbA-trnH*), and *Photosystem II K protein—I spacer* (*psbK-I*) have been proposed (Lahaye et al., 2008; Ghorbani et al., 2017; Mallott et al., 2018; Thakur et al., 2019). Similarly, the presence of disruptions due to differences in the demography of species, or rare but recorded events in which different species share the same haplotype, generate the need for new strategies, such as the combination of markers and the evaluation of different regions (Xiao-Xian and Zhe-Kun, 2007; CBOL Plant Working Group, 2009; Pang et al., 2012; Wang et al., 2017; Mallott et al., 2018).

Since the majority of these markers (*matK*, *rbcL*, *trnL*, *psbA-trnH*, *psbK-I*, *and rpoB*) have been studied in specific plant families, their potential for general taxonomic classification is still unknown, or their capacity to discriminate individual species within a complex mixture (metabarcoding), for example, when processing fecal or soil samples (Lahaye et al., 2008; Gillespie et al., 2009; Seberg and Petersen, 2009; Nicolalde-Morejón et al., 2010; De Groot et al., 2011; Korotkova et al., 2011; Diekmann et al., 2012; Gere et al., 2013; Lee et al., 2017). Therefore, emerging strategies combining markers, such as *matK* + *rbcL* could represent a better approximation for some plant groups species; nevertheless, the evaluation of genes distantly located, or that requires multiple amplicons, are not suitable for metabarcoding analysis since it is currently impossible to link both amplicons to a given origin or DNA molecule.

For any given marker, as important as the intrinsic genetic variability is the availability of a tool that will detect it and be able to assign it accurately and precisely to a taxonomical level. One such tool that is widely used for pattern recognition in DNA sequences and serves as a standard when evaluating new classification approaches is the Naïve Bayesian Classifier (NBC) (Busia et al., 2019). NBC is a machine learning technique that generates a supervised classification model based on a training set. Given that NBC assumes that the input variables are independent and have an equal effect on the classification outcome, each variable (or parameter) must be learned by the classifier from the training set, allowing it to form a posterior probability of assignment or classification. This simple model allows for the evaluation of huge datasets. Moreover, the effectiveness of this classifier has been demonstrated in applications, such as text classification, medical diagnostics, and applications for data administration (Domingos and Pazzani, 1997; Hellerstein et al., 2000). Databases, such as the *Ribosomal Database Project* (RDP), and software, such as *QIIME2* and *MOTHUR* use Bayesian approaches for the taxonomical classification of nucleic acid sequences (Wang et al., 2007; Schloss et al., 2009; [Bibr B14][Bibr B4]; Bolyen et al., 2018).

The use of Bayesian Classifiers in taxonomic classification requires a reference set of DNA sequences with their respective taxonomic labels. Furthermore, sequence classification depends on the type of marker, the training set, and length of the k-mer (Werner et al., 2012). Here, we used six chloroplast gene sequences to evaluate their classification power (*matK*, *rbcL*, *trnL*, *rpoB*, *psbA-trnH*, *and psbK-I*) using an NBC, and to analyze their performance when considering variables, such as marker selection, and representativity in databases. We provided a statistical evaluation of the different marker performance based on the distribution of sensitivity and accuracy (F1 score). Finally, we evaluated genera with low classification performance with the aim to find biological explanations for their misclassification and make some suggestions for researchers who suspect that they have species of these genera in their samples.

## Materials and Methods

### Data Preparation

Six chloroplastic markers (*trnL*, *rpoB*, *rbcL*, *matK*, *psbA-trnH*, and *psbK*) were chosen based on the representation in public databases and frequent use in literature. Sequences were downloaded from GenBank on September of 2018 using as *entrez query*: txid3193 (corresponding to embryophyta), with minimum length of 50 bp (e.g., txid3193[Organism:exp] AND tRNL[Gene Name] AND 50:400000000[Sequence Length] NOT UNVERIFIED [Title]). The same query was used for each of the other markers (*matK*, *rbcL*, *rpoB*, *psbA-trnH*, *and psbK-I*) by replacing the corresponding gene name. Sequences were downloaded in GenBank format and imported into Geneious R9 (Biomatters, New Zealand). Features (genes) were extracted in FASTA format using the “extract annotations” feature of Geneious. The taxonomic distribution of each marker is represented in Table 1. The accession numbers of the six chloroplast markers are available in Supplementary Table 1.

**TABLE 1:** Taxonomical representation of selected markers at different taxonomical levels.

Barcodes	Species	Genus	Family	Order	Class	Phylum
*rbcL*	30,208	8,151	670	136	17	1
*matK*	26,382	6,377	483	113	13	1
*trnL*	22,027	5,023	451	116	13	1
*psbA-trnH*	5,059	1,102	204	78	12	1
*rpoB*	3,996	1,736	305	94	12	1
*psbK*	3,579	1,465	249	88	11	1

*The number of different entities at each taxonomic level for each marker gene is shown.*

### Extraction of Taxonomic Information From Gene Markers

The accession number of each sequence was used to download the corresponding taxonomic information from the National Center for Biotechnology Information (NCBI) taxonomy. Taxonomic information per sequence was organized into the corresponding taxonomic levels: Phylum (P), Class (C), Order (O), Family (F), Genus (G), and Species (S). This was done using in-house scripts wrapped around the software ETE (Huerta-Cepas et al., 2016). The taxonomic information was assigned to every sequence following the pattern of the Greengenes database for bacterial classification (DeSantis et al., 2006). The sequences went through two independent filters: first, maintaining only those that had complete taxonomic classification, as reported in NCBI Taxonomy DB; second, keeping those with almost full-length for the corresponding marker. Sequence number variation after every filter is available in Supplementary Table 2. Size filtering was performed in Geneious.

Finally, we balanced the dataset to reduce possible biases in the data (over- or under-representation of certain species). Each DNA marker was balanced according to the number of sequences present at the species level. Two different datasets were generated for each molecular marker: the first one with species having a minimum of two sequences and a maximum of 20 sequences per species (the dataset 2–20), and the second one having a minimum of five sequences and a maximum of 20 sequences per species (the dataset 5–20). For both cases, species that had more than 20 sequences were randomly subsampled to 20 sequences. On one hand, the 5–20 dataset was generated to evaluate the performance of every marker at different taxonomic levels. On the other hand, the 2–20 dataset was only used to determine how sensible does the model is for underrepresented taxons (i.e., species with only 2–4 sequences available). The script used to perform this balancing is available in https://osf.io/qtz59/?view_only=538ab7719073498abfaea0ab1b29d2ba.

### Classification Algorithm

We used the NBC as implemented in MOTHUR (Schloss et al., 2009). This implementation follows the algorithm described by Wang and collaborators (Wang et al., 2007). Here we used the script classify.seqs from MOTHUR which requires two input files, one with the sequences to build the classification model and another with the full taxonomy for each input sequence separated by each taxonomic level. As output, it will generate a classification with the name of the sequence and a full taxonomic classification with the bootstrap value obtained at each level.

To the best of our knowledge, the algorithm, as described by Wang and collaborators, take each sequence from the dataset, and decompose them into a vector of words of certain size *k* (k-mers), size 8 by default, generating a vector of k-mers and their corresponding abundances. Those vectors are used to calculate a joint probability and hence a probability of assignment for any new sequence (Wang et al., 2007). Confidence estimation of the assignment is implemented in MOTHUR as well. For this, for every sequence that is going to be classified, one hundred (100) random subsets of 1/8 of the k-mer set are chosen randomly and the resulting vector is used for classification. The confidence estimation is then the number of times (out of 100) or bootstrap that the given assignment is obtained. Traditionally an 80% bootstrap value has been selected as having a high precision and accuracy and hence, it was selected as a threshold for subsequent analyses and only results with classifications above that threshold were considered as “trusted” assignments.

As a cross-validation methodology, we chose the “leave-one-out” method (Wang et al., 2007), as it is one of the most exhaustive cross-validation methods. This method is implemented in MOTHUR in the script classify.seqs. Briefly, one sequence is extracted from the dataset, the remaining sequences are used to train the Bayesian model and then the extracted sequence is classified against the recently trained model. This procedure was repeated for each sequence in the dataset and for all markers. The resulting classification for each of the “left out” sequences were then used to calculate the accuracy and precision of the models. The scripts used to run MOTHUR and the final trained model generated are available here https://osf.io/qtz59/?view_only=538ab7719073498abfaea0ab1b29d2ba.

### Statistical and Graphical Analysis

The program RStudio version 1.1.456 (RStudio Team, 2015) was used for graph generation and statistical analysis. The caret package (Kuhn, 2008) was used for the calculation of F1 score. The F1 metric was chosen because it represents the balance between correct and incorrect classifications (precision and recall). Precision is defined as the number of True Positives divided by the number of True Positives plus the number of False Positives. Recall is the number of True Positives divided by the number of True Positives plus the number of False Negatives. F1 score = Precision/Recall. A model that performs perfectly would have an F1 score equal to 1, whereas a model that performs poorly would have a score toward zero (0). An important aspect to highlight the use of F1 score is that it ignores the true negatives which in this type of classifiers are usually the large majority and would bias the values obtained. A confusion matrix was constructed using the real and predicted taxonomic assignments for the 5–20 dataset. All data and calculations are available in Supplementary Table 3. The F1 score for a given genus was calculated as the average value for all the species within the genus. This information was represented on a heatmap. We selected genera with the lowest classification scores—F1 (<0.25)—to explore possible reasons that may be responsible for the constitutive misclassification of the sequences. A multiple sequence alignment (MSA) was generated using MUSCLE V3.81 (Edgar, 2004). The graph was elaborated using the graphic tool for alignment evaluation AliView (Larsson, 2014). The R-package ggplot2 was used for the graphical representation of F1 score (Wickham, 2016).

## Results

### Dataset Exploration

In this study, we used two different datasets (2–20) and (5–20) to determine marker performance and dataset bias, respectively, for plant classification in metabarcoding. The taxonomic distribution is shown in Table 1. Sequence and species count for both 2–20 and 5–20 datasets are presented in Table 2, showing the highest number of genera and species for markers *trnL*, *rbcL*, and *matK*. Moreover, *rbcL* was the marker with the highest number of sequences and species available in databases after quality control and filtering (Table 2, 2–20 dataset). Species with just one sequence as representative is impossible to be used for training a model. It is important to notice the high decrease in the number of species due to their representation by one single sequence, about 50% of all the dataset for most markers was lost in the filtering process (Supplementary Table 2).

**TABLE 2:** Number of species and sequences in the datasets used in this study.

Barcodes	2–20		5–20	
	# of species	# of sequences	# of species	# of sequences
*rbcL*	9,119	27,836	1,125	8,405
*matK*	7,732	24,413	946	7,998
*trnL*	5,218	19,355	919	8,861
*psbA-trnH*	1,744	6,838	370	3,400
*rpoB*	2,721	6,667	102	872
*psbK*	2,694	6,589	99	850

### Distribution of Bootstrap Values

**FIGURE 1:**
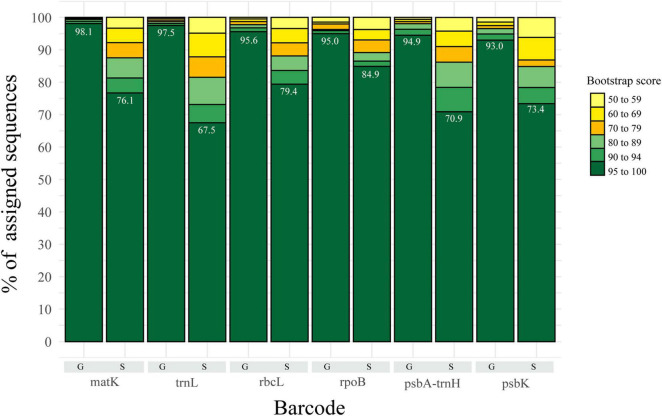
Representation of the percentage of assigned sequences (i.e., classified) by the Bayesian Classifier at species and genus level for the 5–20 datasets. Shades of green represent the fraction of sequences with bootstrap values greater than 80 (proposed threshold for accuracy). Yellow represents the fraction of sequences with bootstrap values lower than 80, thus not used for further analysis.

Classification with the NBC was used as implemented in MOTHUR and described in methods, for both datasets at all taxonomic levels. At higher taxonomic levels, classification was more accurate, with a decrease in accuracy at lower levels, such as genus and species. At the genus level, most of the assigned sequences exhibit bootstraps values with over 90% value, regardless of the marker used, whereas for species, confidence of the classification decreased, with values in the (60–69) and (70–79) range, with *trnL* as a good example (Figure 1). However, the largest category corresponded to those sequences assigned with >95% bootstrap with both datasets (Figure 1 and Supplementary Figure 1). Here, we used a cut-off bootstrap value of 80% for evaluation of the taxonomical assignments.

### Marker Performance and Dataset Bias

**FIGURE 2:**
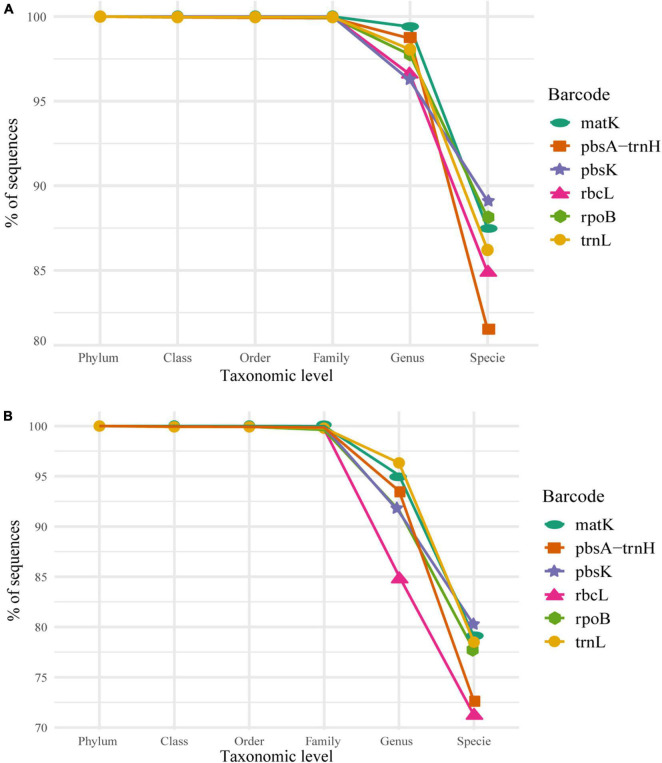
Individual marker performance, as percentage of correctly classified sequences, of the Naïve Bayesian Classifiers (NBC) from Phylum to Species based on the selected bootstrap criterion (80%) in **(A)** the 5–20 dataset and **(B)** the 2–20 dataset. Consider that the scale for the *y*-axis in both plots varies, 80–100% for panel **(A)** and 70–100% for panel **(B)**, for visualization purposes.

We tested the accuracy at different taxonomic levels. The average accuracy of phylum, class, order, and family for every marker, for both datasets (the 5–20 and 2–20), was above 98% of correctly assigned sequences (Figures 2A,B). This implies a similar classification power for all the markers at these taxonomic levels. It is worth highlighting the fact that our final dataset contained only one phylum, thus, classification at this taxonomic level could be biased due to the absence of other phylum. At the genus and species level, a sharp decline in correct assignments was observed, with genus at about 90% and species at about 80% of correctly assigned sequences for the 5–20 dataset (Figure 2A). There was a significant difference in the performance between the 5–20 vs. 2–20 datasets, with the former displaying a better performance at genus and species levels (95 and 80%, respectively; Figure 2A), than the latter (85 and 70%, respectively; Figure 2B) verifying the impact of underrepresented taxons in the classification power of the algorithm. In consequence, we selected the 5–20 dataset for subsequent analyses.

**FIGURE 3:**
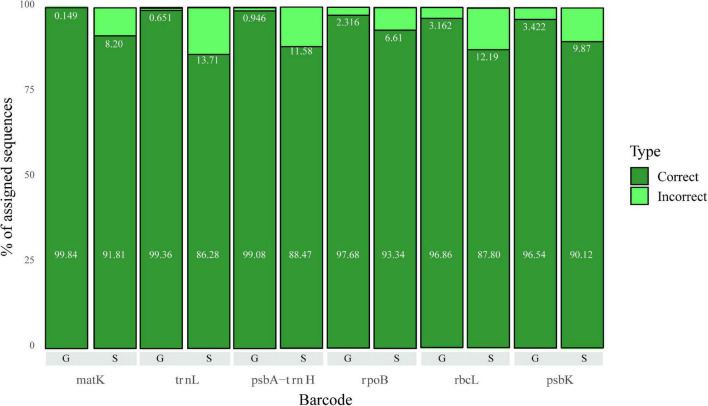
Correct and Incorrect classifications for Genus (G) and Species (S) levels for all markers evaluated in the 5–20 dataset. The value corresponding to the percentage of correct assignments is shown in the bottom of the bar, while the value for incorrect assignments is right below the corresponding light green bar.

We evaluated the performance of each marker at the genus and species level (Figure 3). We observe that *matK* and *trnL* are the markers with the highest rate of correct assignments at the genus level. For species, *matK* and *rpoB* seems to be the best markers (fewer incorrect assignments); however, at this level none of the markers surpasses 95% assignment accuracy (the number of sequences per marker is represented in Table 2).

### Using Classifiers on Problematic Genera

**FIGURE 4:**
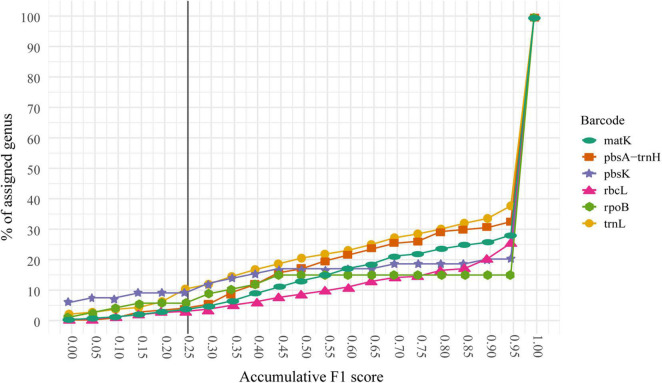
Cumulative F1 score for the 5–20 dataset for every marker. The cumulative percentage of assigned genus at every F1 score threshold is shown. Thus, 100% of the genera are assigned at a cut-off of 1.0 or below. Notice that less than 10% of the genera had an F1 score of 0.25 or below and 65% of the genera shows a score of 0.9 or above for every marker. The shown line defines the threshold for the genera that will be handpicked to evaluate their possible causes of misclassification.

In general, fewer than 15% of the sequences were misclassified at the species level. Furthermore, those misclassified sequences tend to belong to a reduced number of genera. Hence, we decided to identify the genera with the lowest classification performance at the species level to determine potential reasons for their problematic classification. We used the F1 score for the selection of those problematic genera. For a correct interpretation of the F1 score, it is necessary to remember that the closer the score is to 1, the higher the quality of the prediction generated by the precision value (proportion of sequences correctly classified or true positives) and the recall (proportion of sequences belonging to one category and classified as another or false positives). The respective cumulative percentage of genus classification for every marker based on the F1 score is shown in Figure 4 and Supplementary Table 3. Essentially, we found three different scenarios:

(a) The genus has one or multiple markers and at least one performs well: these genera (*n* = 684 or 65%) have an F1 score greater than 0.25 in one or multiple markers. However, there are some cases (*n* = 34 or 3.21%) where the genera have an F1 score below 0.25 for at least one marker but were able to be classified correctly by at least one another marker.

(b) The genus has only one marker and it performs poorly. These genera (*n* = 36 or 3.4%) have only data (sequences) available for one marker and the F1 score ≤ 0.25. In this case, there was no further information to evaluate these genera.

(c) The genus has multiple markers and none of them performs well: There are genera (*n* = 7 or 0.66%) with multiple markers and with F1 score < 0.25 in all of them constituting an important dataset for further analysis into the possible causes for the misclassification. We call these genera “problematic genera.”

**FIGURE 5:**
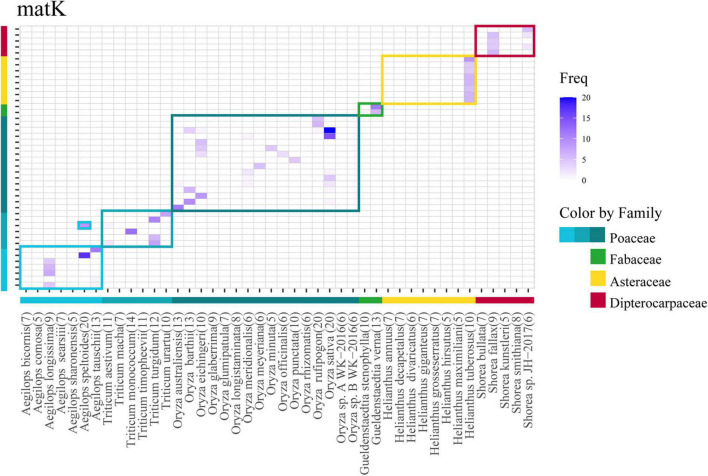
Heatmap corresponding to the confusion matrix of assignment for problematic genera in the case of the *matK* marker. Species from selected genera are depicted in the same order in the *x*- and *y*-axis. Horizontal band color and vertical bar colors at the bottom and side of the heatmap correspond to the families evaluated. Squares enclose their corresponding species. Species in *X*-axis (original) are predicted as one of the *Y*-axis (predicted). The numbers within parenthesis represents the number of sequences for that species.

**FIGURE 6:**
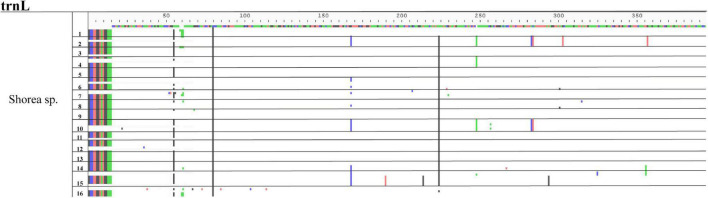
Multiple sequences alignment (MSA) example of species variation from the genus *Shorea* using *trnL* marker. The sequence on the top is the alignment consensus of the genus *Shorea* sp. The different colors below this line indicate the variations within the sequences. The numbers at one side represents the species of this genus. Horizontal lines represent divisions between the different species. Notice that there are no specific variations that could work as specific markers for any given species.

Among the selected problematic genera, seven showed consistent problematic assignments (*Aegilops*, *Gueldenstaedtia*, *Helianthus*, *Oryza*, *Shorea*, *Thysananthus*, *and Triticum*), and no marker was efficient in classifying these taxa. The heatmap in Figure 5 shows an example of the widespread classification problems in these genera for *matK* (the other markers are in Supplementary Figure 2). Most of the misclassification problems occur between species originating from the same genus; however, some misclassifications are seen at the genus level for the family *Poaceae* between *Aegilops* and *Triticum* (Figure 5). This happens for markers *psbK*, *rbcL*, and *rpoB*. Finally, we performed an analysis of problematic genera and their respective species for every marker by a Multiple Sequence Alignment to evaluate if the low performance of the assignment task was correlated with low intraspecific variability. Figure 6 is an example of the genus *Shorea* for the marker *trnL*. As expected, we found very low variation in the multiple alignment within these problematic genera.

## Discussion

The current study shows an evaluation of the most relevant markers used in literature for plant classification, using one of the methods that has demonstrated greater effectiveness of classification in metabarcoding and with the maximum number of sequences that can be obtained in public databases. This process was performed to identify the markers with the highest accuracy at different taxonomic levels for metabarcoding analysis and to identify genera with problematic species. Not surprisingly, *rbcL* was the marker with the highest number of sequences, given that historically it has been the most used marker (Kress and Erickson, 2007; Hollingsworth et al., 2011).

### Dataset Exploration

Our comparative results between the 5–20 and 2–20 datasets show that better performance is achieved with datasets with good representation per class (5–20 dataset), rather than with datasets with a higher number of classes, but lower representation per class. This is true for the most classification methods. It further highlights the importance of increasing the reports of sequences from different taxonomic lineages in public databases, since only a good representation of a given lineage will allow a proper model training for accurate classification.

### Markers Examination by Bayesian Classifiers

Bayesian classifiers have been successfully used in metabarcoding strategies. The method employed here has the added benefit of bootstrapping the k-mers used to reduce the chance of overfitting and guarantee that no single k-mer is responsible for a given classification. Thus, classifications with high bootstrap values show the robustness of the method. In our case, we needed to find a balance between high confidence in the assignment, given by the bootstrap value, and the high percentage of classification. We selected a frequently used 80% bootstrap threshold for our analyses, which allowed the classification of over 80% of the sequences at species level and over 95% of the sequences at the genus level or above. At species level, *matK* and *rpoB* appear to be the best markers (fewer incorrect assignments); however, at this level, none of the markers surpasses 95% of assignment accuracy. This level of accuracy, which is acceptable in other fields of machine learning, is probably too low to be considered a “good classifier” at the species level. According to this result, it is recommended to use markers above the 98% accuracy at the genus level, which are *matK* and *trnL*.

When analyzing the performance of the individual markers, we want to highlight *matK* and its performance on taxonomic assignment, even with datasets of sparse representation, such as the 2–20 dataset. Its outstanding performance could be attributed to the high interspecific variability of the marker (Mankga et al., 2013; Jiménez-Mejías et al., 2016; Elansary et al., 2017). Our results were consistent with the recommendations from other authors based on the low performance of other single markers at the species level (Whittall et al., 2010; China Plant BOL Group, 2011; De Groot et al., 2011; Hollingsworth et al., 2011; Gere et al., 2013; Chen et al., 2016; Kress, 2017; Menezes et al., 2018). Although a proposed alternative for barcoding was the combination of multiple single gene markers, this approach is unsuitable in the context of metabarcoding given that it is currently technically impossible to tie two or more markers to an individual in an environmental sample, unless the markers were next to each other and amplifiable as a single amplicon. Our results indicate that for single marker classification, *trnL* and *matK* are the best choices when classifying up to genus level and are resilient to low sequence representation in databases. In addition, we recommend *matK*, which was the marker with the best performance overall at species level. However, further studies are necessary to determine flanking regions of *matK* that could improve the species classification. For general plant classification, we agree with the recommendation from several authors of using *matK* in combination with other genes (Braukmann et al., 2017; Xu et al., 2018; Li et al., 2021).

### Problematic Genera

For the genera with problematic classifications, we found three types of behavior (Supplementary Table 3). First, we identified some genera (3.2%) with very low F1 score with one specific marker, but with enough resolution for other markers (e.g., *Citrus*, *Adenophora*, *Oenothera*, *Rosa*, and *Vitis*), thus representing a limitation of a given marker-genus pair for classification. Second, there were some genera (3.4%) with information for only one marker, making it impossible to compare or gather more information from those, highlighting the importance of generating more data on those markers for under-represented taxa. Moreover, most of these genera had a low number of sequences which itself implies an associated factor to their low classification accuracy. Third and finally, there was a set of genera (0.66%) with two or more markers generating misclassifications. For this final case, we found a total of seven genera *Aegilops* (*Poaceae*, *goatgrasses*), *Gueldenstaedtia* (*Fabaceae*, *legume*), *Helianthus* (*Asteraceae*, *sunflower*), *Oryza* (*Poaceae*, *rice*), *Shorea* (*Dipterocarpaceae*, *lauan*), *Thysananthus* (*Lejeuneaceae*, *liverwort*), *and Triticum* (*Poaceae*, *wheat*).

A deeper look at these genera allowed important observations. *Gueldenstaedtia* is a small genus of *Fabaceae* that is very similar to the *Tibetia* genus (Xie et al., 2016). Most of the misclassified species in *Gueldenstaedtia* were assigned as *Tibetia* species. We found a similar situation with the genera *Shorea* and *Thysananthus*. *Shorea* is a very important genus related to timber and wood products. Tsumura et al. (2011) reported that some species within *Shorea* have identical sequences for multiple chloroplast regions, indicating that it may be difficult to discriminate between closely related species. In their manuscript, they propose a method for the identification of species of this genus and suggest using other non-chloroplast-based markers, such as *ITS* for the identification of *Shorea*. Finally, for *Thysananthus*, one of the largest genera of liverworts that has been monographed worldwide, some authors suggest that given their morphological overlap, the molecular evidence and the lack of morphological characters separating them from *Mastigolejeunea*, they should be merged as a single genus (Sukkharak and Gradstein, 2017). Thus, the misclassification on those three genera seems to be more related to the lack of biological divergence than the performance of the markers themselves.

The genera *Aegilops* and *Helianthus* showed a pattern that regardless of the species being assigned, all assignments were collapsed to a single species. For *Aegilops*, this pattern was observed in many of the evaluated markers, such as *matK* (Figure 5, notice most species are classified as *Aegilops longissima*). For *Helianthus*, the pattern was presented in *matK*, *rbcL*, *psbK*, and *rpoB* (Supplementary Figure 2). A second pattern of apparent random assignments was found for *Oryza*, *Triticum*, *Citrus*, and *Shorea*, just to mention some of the examples. This behavior was found for more than one marker (*matK*, *rpoB*, *rbcL*, *psbK*, and *psbA-trnH*). Analyzing those genera and their respective species using MSA, we noticed a very low or non-existent variation among the sequences (Figure 6) pointing again at very low biological divergence among the species of the genus, and likely the cause of the low F1 score for those genera.

We identified a particular pattern among *Aegilops* and *Triticum*, both belonging to the *Poaceae* family. In these genera, for different markers, occurs a classification of *Aegilops* species as *Triticum* ones, and vice versa. In the most recent phylogenetic classification of *Poaceae*, these two genera have been assigned as being part of the subtribe Triticinae (Soreng et al., 2015) based on methods of maximum likelihood on *matK* and *ndhF* markers. In those genera, there is evidence of hybridization (Loureiro et al., 2009; Tsunewaki, 2009; Zhang et al., 2010) using allopolyploidization as the major force leading to the diversification during the evolution of *Triticum* species. *Aegilops* has been characterized as a wild relative of *Triticum* (wheat). Cultivated wheats and their close wild relatives belong to the genus *Triticum*, a member of the tribe *Triticeae*, which contains 300 species (Clayton and Renvoize, 1986). Together, this evidence suggests some of the possible causes of classification problems with the assignment of these two genera.

Hollingsworth et al. (2011) suggests seven key factors that may lead to a lower level of success in species discrimination, such as hybridization, polyploidy, life history, breeding systems, species history, level of taxonomic “splitting,” and seed dispersal. In all our problematic genera (*Aegilops*, *Gueldenstaedtia*, *Helianthus*, *Oryza*, *Shorea*, *Thysananthus*, *and Triticum*) those patterns were present. In *Aegilops*, there is evidence of allopolyploid, containing multiple chloroplast haplotypes, each identical to haplotypes of the diploid progenitor species, indicating multiple origins as the major source of variation (Meimberg et al., 2009). In *Triticum*, there was evidence of hybridization and polyploidy. Hybridization occurs between wheat cultivars because mixed cultivation of different wheats with different ploidies is a tradition and still common practice in the Middle East and Transcaucasia. Furthermore, wild wheat species can be involved in hybrid swarms in regions where they naturally grow in and around the areas of wheat cultivation (Matsuoka, 2011). There was a similar pattern with the genus *Oryza* and *Helianthus* given their human domestication and their economic importance as food source (Blackman et al., 2011; Molina et al., 2011; Kantar et al., 2012; Civáň et al., 2015; Badouin et al., 2017; Stein et al., 2018).

### Caveats and Recommendations

Finally, it is important to highlight that the taxonomy source of the evaluated sequences was GenBank. This is a database of primary sequences where the submitter gives the taxonomic assignment of the uploaded sequence and thus, it is prone to human error. A certain error percentage is expected by using such a database. We tried to minimize the error by using several representative sequences per species, but this was limited in some cases.

Our results show that the NBC is a tool that could be used for plant classification. Based on the results of this classifier for the taxonomic levels: class, order, or family, any of the evaluated markers would sufficiently fulfill the expected accuracy and precision. For classification at the genus level, *trnL* and *matK* are the recommended choice due to their high performance of classification, even on taxons with low number of sequenced representatives (2–5 sequences per taxon). We only evaluated classifications based on Naïve Bayesian models; however, other methods using machine learning models, such as support-vector machine (SVM) could be of interest for future validations. Due to the limitations in metabarcoding studies and with long-read sequencing technologies becoming more prevalent, we propose the evaluation of chloroplast regions that contains more than one gene, identical to the regions close to *trnL*, *matK*, *psbA-trnH*, *rbcL*, *and rpoB* using NBC as a modeling method.

Some specific combinations of marker-genus were problematic for classification; however, several of them could be assigned with other markers. We further analyzed the possible reasons of multiple marker misclassification for the genera *Aegilops*, *Gueldenstaedtia*, *Helianthus*, *Oryza*, *Shorea*, *Thysananthus*, *and Triticum*, identifying in all cases that it was likely related to biological conditions, such as hybridization, polyploidy, and evolutionary history, and not due to the algorithm or technical difficulties. Barcodes are powerful tools for sequence classification and plants are no exception. However, only a thorough analysis, such as the one performed in the current study can provide evidence of the usability of the different markers and their limitations. Here, we used the most common molecular markers together with all the available sequences on public databases and a state-of-the-art classification method to determine the best performing marker for each taxon on potential interest and, finally, release a Green Genes-like database to be used by the researchers on their own research.

## Data Availability Statement

The original contributions presented in the study are included in the article/Supplementary Material, further inquiries can be directed to the corresponding authors.

## Author Contributions

AR and JA contributed to the conception, designed of the study, and reviewed the manuscript. JA organized the database. LM-C performed the statistical and classification analysis, wrote the draft, and final version of the manuscript. All authors contributed to manuscript revision, read, and approved the submitted version.

## Conflict of Interest

The authors declare that the research was conducted in the absence of any commercial or financial relationships that could be construed as a potential conflict of interest.

## Publisher’s Note

All claims expressed in this article are solely those of the authors and do not necessarily represent those of their affiliated organizations, or those of the publisher, the editors and the reviewers. Any product that may be evaluated in this article, or claim that may be made by its manufacturer, is not guaranteed or endorsed by the publisher.
