# Supplementary material for: Taxonomical Evaluation of Plant Chloroplastic Markers by Bayesian Classifier
Source: Front Plant Sci. 2022 Feb 3;12:782663. doi: 10.3389/fpls.2021.782663 (PMC8850773; doi:10.3389/fpls.2021.782663)

Supplementary Material

# Supplementary figure 1. Representation of the percentage of assigned sequences (i.e., classified) by the Bayesian Classifier at species and genus level for the 2 to 20 datasets. Shades of green represent the fraction of sequences with bootstrap values greater than 80 (proposed threshold for accuracy). Yellow represents misclassifications, the fraction of sequences with bootstrap values lower than 80, deemed as misclassified.
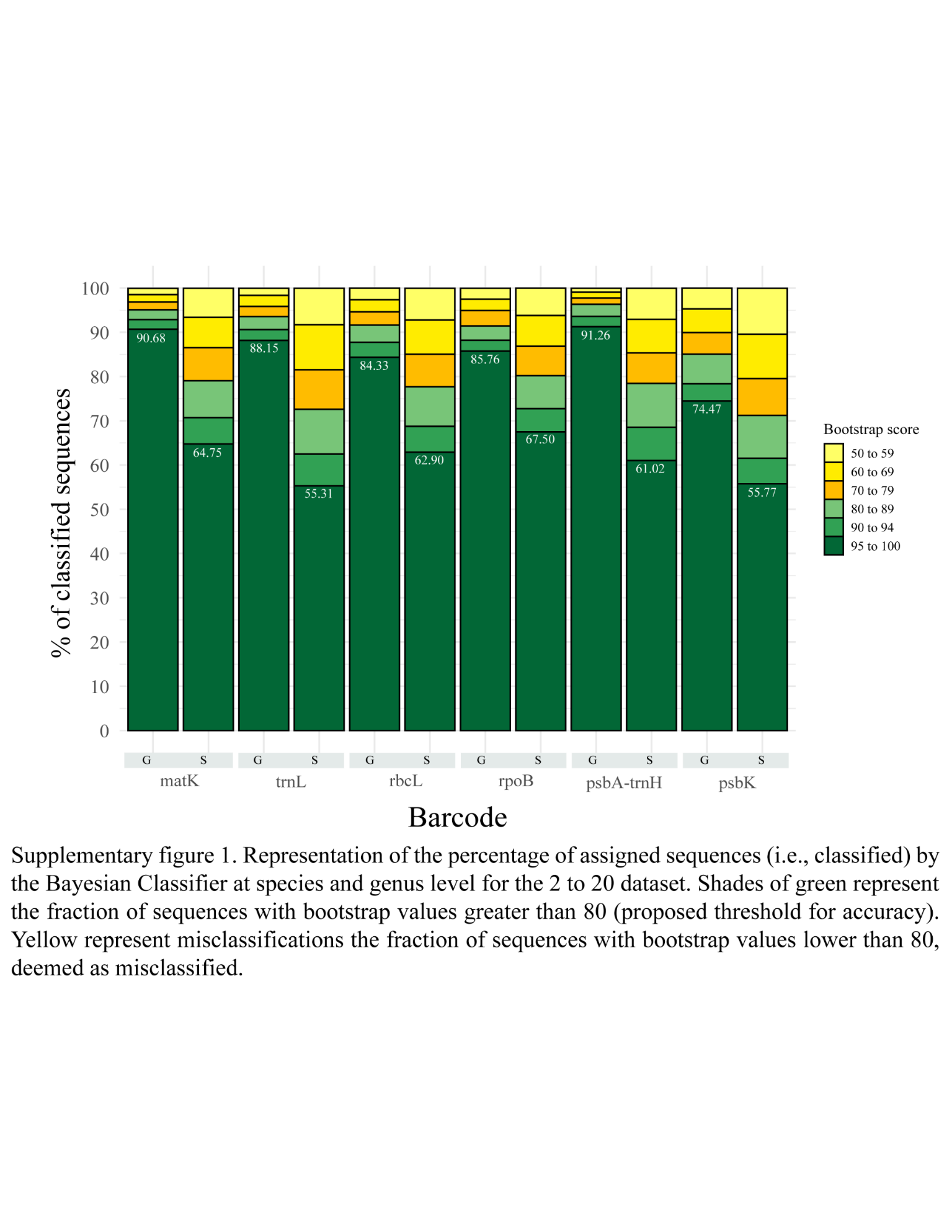


# Supplementary figure 2. Heatmap corresponds to the confusion matrix of assignment for problematic genera in chloroplast marker (A) psbK (B) rbcL (C) trnL (D) rpoB (E) psbA-trnH. Species from selected genera are depicted in the same order in the x and y-axis. Horizontal band color and vertical bar colors at the bottom and side of the heatmap correspond to families evaluated. Squares enclose their corresponding species. Species in x-axis (original) is predicted as one of the y-axis (predicted). The numbers within parentheses represent the number of sequences for that species. The species are shown in the same order in the y-axis.
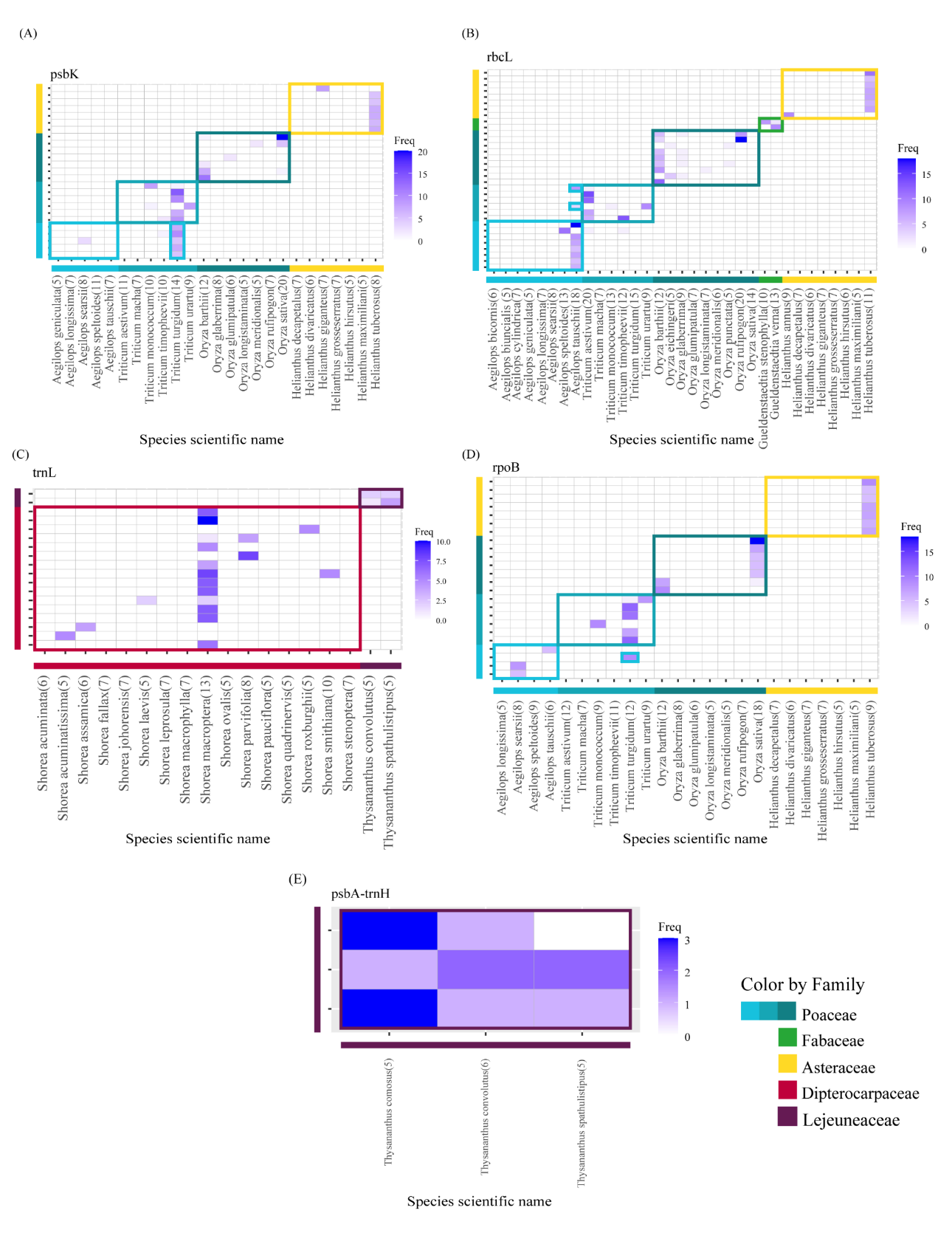

Supplement: Supplementary file 4 [file Data_Sheet_1.docx]
